# Hybrid identification and genetic variation of *Elymus sibiricus* hybrid populations using EST-SSR markers

**DOI:** 10.1186/s41065-017-0053-1

**Published:** 2017-12-12

**Authors:** Xuhong Zhao, Junchao Zhang, Zongyu Zhang, Yanrong Wang, Wengang Xie

**Affiliations:** The State Key Laboratory of Grassland Agro-ecosystems, Key Laboratory of Grassland Livestock Industry Innovation, Ministry of Agriculture, College of Pastoral Agriculture Science and Technology, Lanzhou University, Lanzhou, 730020 People’s Republic of China

**Keywords:** *Elymus sibiricus*, EST-SSR marker, Hybrid identification, Genetic diversity

## Abstract

**Background:**

*Elymus sibiricus* is an important native grass in Qinghai-Tibetan Plateau. Seed shattering is a serious problem for *E. sibiricus*, especially at harvest time. Cross breeding is an effective way to create new varieties with beneficial characteristic or improved traits, and to broaden genetic base.

**Results:**

In this study, we created five hybrid populations by crossing seven *E. sibiricus* genotypes that have seed shattering variation. Then, nine EST-SSR primers were used for hybrid identification based on DNA fingerprinting, and genetic diversity analysis of hybrid populations and their respective parents. A total of 15 hybrids were identified. An analysis of amplified polymorphic bands among genuine hybrids and their respective parents revealed mainly two types of markers: 1) hybrids shared bands exclusively amplified in both parents; 2)hybrids shared bands exclusively amplified in male parents. For each hybrid population, the total number of amplified bands ranged from 37 to 57, the percentage of polymorphism varied from 65.12% to 75.68%, with an average of 70.51%. Novel bands found in each hybrid population varied from 0 to 9 bands, suggesting an occurrence of rearrangements in the hybrid population. The structure analysis revealed that all hybrid populations and parents were assigned to eight groups. The principal coordinate analysis (PCoA) showed similar results.

**Conclusions:**

In general, this study proved EST-SSR markers are efficient for hybrid identification, and suggested more genetic variation could be captured in hybrid populations by crossing breeding.

## Background

*Elymus sibiricus* L., named also siberian wild rye, is a perennial, caespitose, gramineous *Elymus* forage grass [1], indigenous to Northern Asia [2]. Its natural geographic distribution extends from Sweden to Japan and even to parts of Alaska and Canada [3]. It is wildly utilized in cultivated pasture and natural grassland in north China, owing to its excellent tolerance to low temperature and drought, and good forage quality and palatability [4].

In recent years, overgrazing and climate change resulted in grassland degeneration, it has created a need for revegetation/restoration of rangeland ecosystems in north China. As a native grass in north China, *E.sibiricus* have the potential to be used in degenerated grassland due to its good adaptability to local environment. However, few *E.sibiricus* cultivars are available for revegetation /restoration projects in these regions. Until now, progress in cultivar development and improvement in *E.sibiricus* has seriously lagged behind crop plants even other forage grasses. In the last 20 years, only 6 cultivars have been developed from wild materials [5]. Meanwhile, this species has serious problem with seed shattering. In previous study, we have identified some low seed shattering genotypes from wild *E.sibiricus* germplasm originated from northeastern Qinghai-Tibet Plateau [4]. These materials are valuable genetic resources for seed shattering improvement in future breeding program. Cross breeding is an effective way to create new varieties with beneficial characteristic or improved traits, and to broaden genetic base of *E. sibiricus* [6]. Generally, these resynthesized breeding materials are genetically diverse from inbred line/cultivar [7], and might have higher heterosis than their parents [8]. Traditionally, we identify hybrids and analyze genetic diversity through morphological traits such as plant height, inflorescence, leaf shape, etc. The process of identification is time-consuming, and the accuracy of morphological identification is also limited due to the influence of environment factors. Compared to conventional hybrid identification through morphological difference in the course of plant breeding, marker-assisted selection (MAS) is more efficient way as it is reproducible, reliable, and independent from environmental conditions, it therefore could be used to mirror directly genetic diversity [9].

Among the available molecular markers, SSRs (simple sequence repeats) or microsatellites, offer an important DNA marker system for hybrid purity testing because of their co-dominance, reproducibility, robustness, and multi-allelic nature [10]. The characteristics of co-dominance give enormous advantages to SSR marker, which can be more clearly to present the band feature of parent plant and their offspring in plant breeding [11]. SSR markers have been widely used to assess hybrid purity in maize [12], rice [13], sunflower [14], cabbage [15], bunching onion [16], cauliflower [17] and some forage grasses like orchardgrass [18]. In addition, SSR markers have been successfully used to distinguish *E. sibiricus* and *E. nutans* originated from Qinghai-Tibet Plateau [19] and detect genetic diversity and variation of *E. sibiricus* accessions worldwide [20]. There are no previous reports of hybrid identification using EST-SSR markers. Genetic information on hybrid populations is limited in *E.sibiricus*.

In this study, we used EST-SSR markers to identify hybrid based on specially amplified DNA fingerprinting and analyze genetic diversity of five hybrid populations and their respective parents in *E. sibiricus*. This study will help lay a foundation for future *E. sibiricus* breeding program.

## Methods

### Plant materials

A total of seven *E. sibiricus* accessions and their offsprings were used in this study (Tables 1 and 2). These seven accessions had different geographical origins, they were genetically and morphological divergent. According to previous genetic diversity study these accessions were clustered to different groups based on SCoT markers [20]. In addition, their selection was also based primarily on several contrasting agronomic traits: seed shattering, plant height, panicle length, etc. *E. sibiricus* is not an endangered or protected species, thus, no permission was required for collecting these samples in China. Single genotype from each parental accession was used to generate the F_1_ hybrids by hand pollination in June 2014. A total of 15 F_1_ individuals were derived from five pairs of cross (Table 2). F_1_ seeds were harvested from the female parents in August 2014. The F_1_ individuals were grown in a greenhouse at approximately 22 °C and a 16 h photoperiod until they were 8 weeks old. The F_1_ individuals were then transplanted to the field in the campus experimental station at Lanzhou University, Yuzhong, Gansu, China (103°34′ E, 35°34′ N). No any permissions were required to carry out field experiment. A total of 15 F_1_ plants were tested by EST-SSR markers to confirm their hybrid status.

**Table 1:** The origin and morphological traits of parents used in this study

Parents	Status	Origin	Morphological characteristics
Y1005-1	Wild	Ruo ergai, Sichuan, China	High seed shattering
LQ03-1	Wild	Luqu, Gansu, China	Medium seed shattering
PI 348916	Wild	Alaska, United State	Low seed shattering, Late flowering
XH09-4	Wild	Xiahe, Gansu, China	High seed shattering
ZhN06-1	Wild	Zhuoni, Gansu, China	Low seed shattering
ZhN06-11	Wild	Zhuoni, Gansu, China	Low seed shattering, tall, long panicle
Chuancao No. 2	Cultivar	Hongyuan, Sichuan, China	Tall, early flowering, medium seed shattering

**Table 2:** Parents and hybrid populations used in this study

Population	Female parents	Male parents	F1 individuals
1	ZhN06-1	Y1005-1	1-H1
2	Y1005-1	ZhN06-11	2-H2, 2-H3, 2-H4, 2-H5
3	ZhN06-11	Y1005-1	3-H1, 3-H2 3-H3, 3-H4, 3-H5
4	XH09-4	LQ03-1	4-H1, 4-H2, 4-H3
5	Chuancao No. 2	PI348916	5-H1, 5-H2

### DNA extraction and PCR amplification

Genomic DNA was extracted from parental plants and individual hybrid plant tissue using SDS (sodium dodecyl sulfate) method [21] (Shan et al., 2011). DNA quantity and quality were verified using a Nanodrop spectrophotometer (NanoDrop Products, Wilmington, DE, USA) and agarose gel electrophoresis. Finally, the DNA concentration was adjusted with ddH_2_O to 25 ng/μL and stored at −20 °C prior to PCR amplification. A total of nine selected polymorphic EST-SSR primers were used for genotyping (Table 3). These primers had been used in a previous genetic diversity study in *E. sibiricus* [4]. The optimal reaction system for *E.sibiricus* was as follows: 2 μL 25 ng/μL DNA, 7.5 μL 2× Reaction Mix (Tiangen Beijing, China), 0.5 μL 10 μM forward primer, 0.5 μL 10 μM reverse primer, 0.2 μL (2.5 U/μL) Golden DNA Polymerase (Tiangen Beijing, China), and 4.3 μL of sterile ddH_2_O in a total of 15 μL reaction volume. PCR amplification was carried out as described by Xie et al. [4]. Then amplification fragments were separated on 6% denatured polyacrylamide gels. After electrophoresis, the gel was stained by AgNO_3_ solution. Then gel was photographed by a Gel Doc (TM) XY System (Bio-Rad, Hercules, CA, USA).

**Table 3:** The 9 EST-SSR primers used in this study

Primer name	Forward primers	Reverse primers
Elw1420s081	GGATAGACCCATGAGCTGACTGAT	CTTTCTCCACAAGTTGAACACAACA
Elw3545s194	CAGCACTAGTATCCACCTCCACCT	TGTTACAGCCTCTTCAGGCTCTTC
Elw5627s404	AGATGAAGCTGGTAACCGAGACAG	ATTTCCTCTAATGGAAGCTCTGGC
Ps1830	GACTCGGCGAAAGGACTCTCT	CTCGACGTCCTTCATGAGCTT
Ps2283	GCCACAACAAGAGAAGACCTTGC	GACCTGCATGATGCTCTCGC
Ps3577	CATCTTGCATATAGCTCCTTCGCT	CTCAAGAAACCCACAATCCAATTC
Ps938	TTGCTCCTATGGTTCCACGTAGTT	AAAGTGAAATTCTGCCATCAGAGC
Ltc0055	AAGAAGAAGAGGCCGAGGAATAAA	CGTGGATGTGCTGCAGGTAGTA
Ltc0157	GCAATGAACACTGAATCAATCGAG	CGTGTGAGACTCATCGATGTTACC

### Data analysis

The amplified bands were scored as present (1) or absent (0), and only reproducible bands were considered. STRUCTURE v2.3.4 software was used to analyze the population structure of *E. sibiricus* accessions and hybrid populations, with the ′admixture mode′, burn-in period of 10,000 iterations and a run of 100,000 replications of Markov Chain Monte Carlo (MCMC) after burn in [22]. For each run, 10 independent runs of STRUCTURE were performed with the number of clusters (K) varying from 1 to 8. Mean L (K) and delta K (ΔK) were estimated using the method described by Evanno et al. [23] To detect genetic relationship among different accessions, a principal coordinate analysis (PCoA) was constructed based on Jaccard′s genetic similarity matrix using DCENTER module in NTSYS (version 2.10), which is more informative regarding distances among major groups [24]. *E. sibiricus* hybrid identification was carried out according to a method used in orchardgrass [18]. Single primer pairs or primer combinations that were diagnostic for parental plants and hybrid plants were used.

## Results

### EST-SSR marker transferability

EST-SSR markers previously developed from Snake River wheatgrass (*Elymus wawawaiensis*), thick spike wheatgrass (*Elymus lanceolatusd*), bluebunch wheatgrass (*Pseudoroegeneria spicata*) and *Leymus* species were used in this study. Finally, a total of 9 primers were selected and used for hybrid identification and genetic diversity analysis, of which 3 from *Elymus* (Elw hereafter), 4 from *Pseudoroegneria* (Ps hereafter) and 2 from *Leymus* (Lt hereafter). The results proved that all 9 primers can be successfully amplified across 22 *E.sibiricus* plants used in this study, with 100% the transferability rate.

### Hybrid identification

**Fig. 1:**
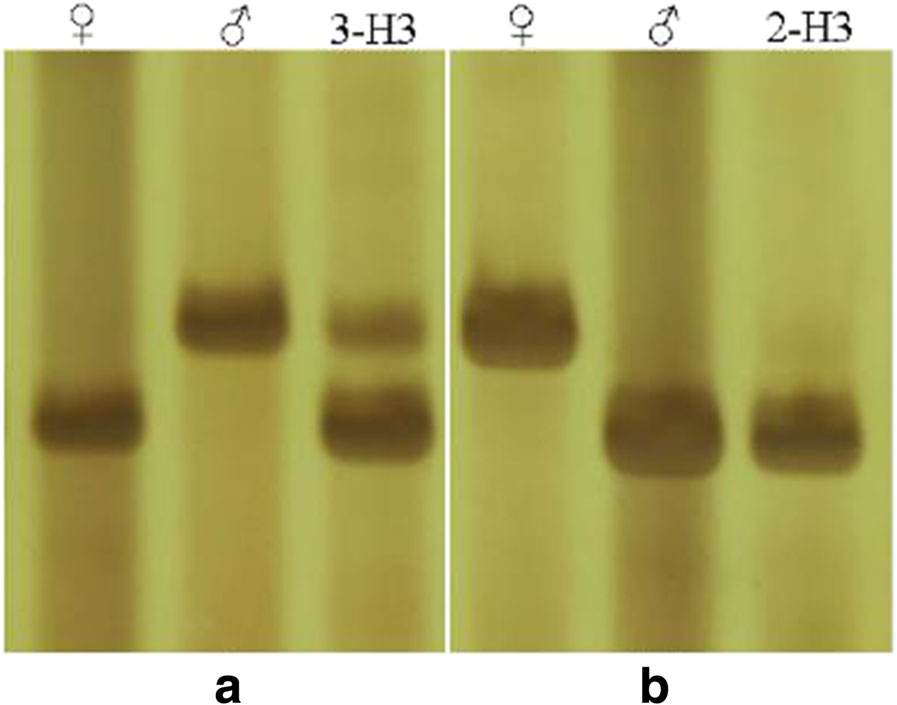
EST-SSR profiles produced with different primers in hybrid and their respective parents. **a**: hybrids shared bands exclusively amplified in both parents; **b** hybrids shared bands exclusively amplified in male parents

**Fig. 2:**
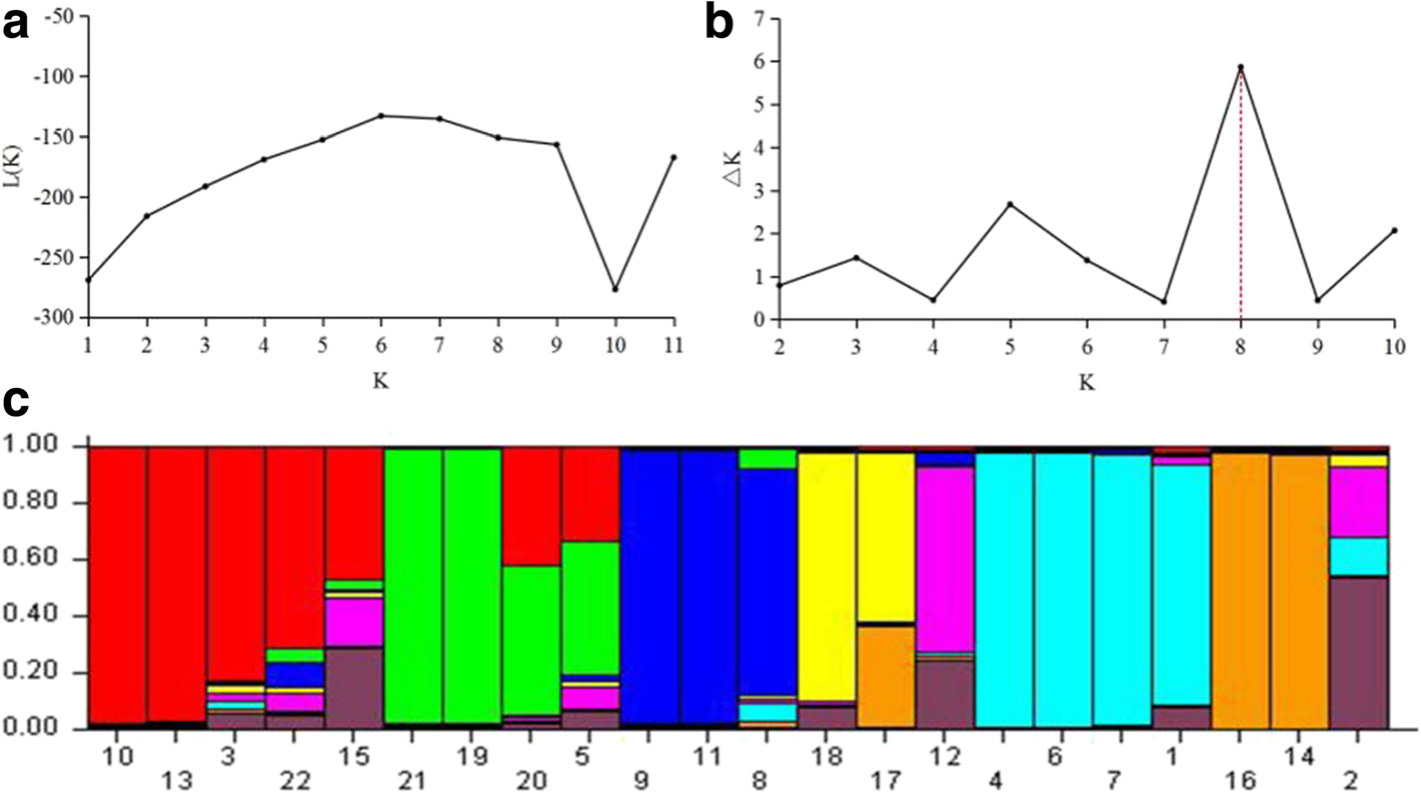
Eight groups of 22 *E. sibiricus* accessions inferred from STRUCTURE analysis and the description of detected the optimum value of K by using graphical method. **a** Mean L (K) over 20 runs for each K value; **b** Maximum delta K (4 K) values were used to determine the uppermost level of structure for K ranging from 2 to 10, here K is 8 and eight clusters; **c** The vertical coordinate of each group indicates the membership coefficients for each accession. 1-22 represented respectively: ZhN06-1, Y1005-1, 1-H1, Chuancao No.2, PI348916, 5-H1, 5-H2, ZhN06-11, 3-H1, 2-H2, 3-H2, 3-H3, 2-H3, XH09-4, LQ03-1, 4-H1, 4-H2, 4-H3, 2-H4, 3-H4, 3-H5, 2-H5

Single primers or primer combinations that were diagnostic for parental plants and hybrid plants were used. In the study, an analysis of amplified polymorphic bands among genuine hybrids and their respective parents mainly revealed two types of markers: 1) hybrids shared bands exclusively amplified in both parents (Fig. 1a); 2) hybrids shared bands exclusively amplified in male parents (Fig. 1b); Finally, according to this method, 15 hybrids were successfully identified using different primers and then used for the genetic diversity analysis (Table 2).

### EST-SSR polymorphism and genetic relationship in hybrid populations and parents

In this study, 9 primers were used for genetic diversity analysis among five hybrid populations and their parents (Table 4). The total number of amplified bands ranged from 37 (Pop 1) to 57(Pop 2, 3), the percentage of polymorphism varied from 65.12 (Pop 5) to 75.68% (Pop 1), with an average of 70.51%.

**Table 4:** EST-SSR results achieved in hybrid populations and their parents

Population	Primer	T	M	TP	MF1	FF1	EF1	PPB (%)
1	Elw1420s081	3	0	3	0	1	0	100.00
	Elw3545s194	3	3	0	3	3	0	0.00
	Elw5627s404	14	0	14	3	2	6	100.00
	Ps3577	4	2	2	2	2	1	50.00
	Ps938	7	1	6	1	1	0	85.71
	Ltc0055	6	3	3	3	3	0	50.00
	Total	37	9	28	12	12	7	75.68
2	Elw1420s081	6	1	5	1	1	4	83.33
	Elw3545s194	3	1	2	1	2	0	66.67
	Elw5627s404	14	2	12	4	5	3	85.71
	Ps1830	9	4	5	5	5	0	55.56
	Ps2283	2	1	1	1	1	1	50.00
	Ps3577	4	2	2	3	2	0	50.00
	Ps938	5	2	3	5	2	0	60.00
	Ltc0055	3	3	0	3	3	0	0.00
	Ltc0157	11	3	8	4	3	1	72.73
	Total	57	19	38	27	24	9	66.67
3	Elw1420s081	5	1	4	1	1	3	80.00
	Elw3545s194	3	1	2	1	2	0	66.67
	Elw5627s404	14	1	13	5	2	3	92.86
	Ps1830	10	3	7	4	2	0	70.00
	Ps2283	2	1	1	1	1	1	50.00
	Ps3577	4	2	2	3	3	0	50.00
	Ps938	5	1	4	1	2	0	80.00
	Ltc0055	3	3	0	3	3	0	0.00
	Ltc0157	11	3	8	3	4	1	72.73
	Total	57	16	41	22	20	8	71.93
4	Elw3545s194	2	2	0	2	2	0	0.00
	Elw5627s404	7	1	6	1	4	0	85.71
	Ps1830	7	2	5	2	6	0	71.43
	Ps2283	3	1	2	1	2	0	66.67
	Ps3577	8	1	7	2	3	0	87.50
	Ps938	6	0	6	0	1	0	100.00
	Ltc0055	3	3	0	3	3	0	0.00
	Ltc0157	5	1	4	1	2	0	80.00
	Total	41	11	30	12	23	0	73.17
5	Elw1420s081	3	0	3	0	1	0	100.00
	Elw3545s194	3	3	0	3	3	0	0.00
	Elw5627s404	6	0	6	0	3	1	100.00
	Ps1830	8	2	6	2	3	2	75.00
	Ps2283	3	0	3	1	1	1	100.00
	Ps3577	4	2	2	2	2	0	50.00
	Ps938	4	1	3	1	2	2	75.00
	Ltc0055	4	4	0	4	4	0	0.00
	Ltc0157	8	3	5	3	5	1	62.50
	Total	43	15	28	16	24	7	65.12

T = Total number of amplified bands; M = Number of monomorphic bands; TP = Total number of polymorphic bands; MF1 = Bands shared by male parents and hybrids; FF1 = Bands shared by female parents and hybrids; EF1 = Bands exclusively present in hybrids; PPB = Percentage of polymorphic bands

Regarding the parental origin of the amplified bands inherited by hybrid populations, similar percentage of parental origin were found in three hybrid populations. The percentages in the first three populations didn’t reach 50%. For example, in the population 1, 32.43% were inherited from ZhN06-1 and 32.43% were from Y1005-1. In the population 2, 47.37% were from ZhN06-11 and Y1005-1 had 42.11% origin. In the population 3, 38.59% were inherited from male parent and 35.09% from female parent. However, in the population 4 and 5, 56.09% and 55.81% amplified bands were inherited from male parents, respectively. Some types of polymorphism evidenced an occurrence of rearrangements in the hybrid populations that resulted from the gain of novel bands (not seen in parental genomes). Total number of bands that exclusively present in hybrid populations ranged from 0 (Pop4) to 9 (Pop2), with an average of 6.2.

The population structure of hybrid populations and their parents was analyzed in this study. Based on maximum likelihood and delta K (ΔK) values, the number of optimum groups was eight (Fig. 2). All accessions were assigned to eight groups. group1 included LQ03-1, 1-H1, 2-H2, 2-H3, 2-H5; group 2: PI348916, 2-H4, 3-H4, 3-H5; group 3: ZhN06-11, 3-H1, 3-H2; group 4: 4-H2, 4-H3; group 5: 3-H3; group 6: ZhN06-1, Chuancao No.2, 5-H1, 5-H2; group 7: XH09-4, 4-H1; group 8: Y1005-1. Every group was mixed genetic ingredient of other groups in different extent. Especially, LQ03-1, PI348916, ZhN06-11, 3-H3, 1-H1 and 2-H5 had more complicated genetic constitutes than the other accessions in the study. 1-H1, 2-H5 and 3-H3 were the F_1_ individuals from population 1, 2 and 3, respectively. However, some accessions presented a purer genetic ingredient within their groups. 4-H1, 5-H1 and 5-H2, the F_1_ plant of population 4 and 5, had same genetic constitutes to female XH09-4 and Chuancao No.2 respectively. The rest of individuals for pure genetic constitutes included 2-H2, 2-H3, 2-H4, 3-H1, 3-H2 and 3-H5, they came from population 2 and 3.

**Fig. 3:**
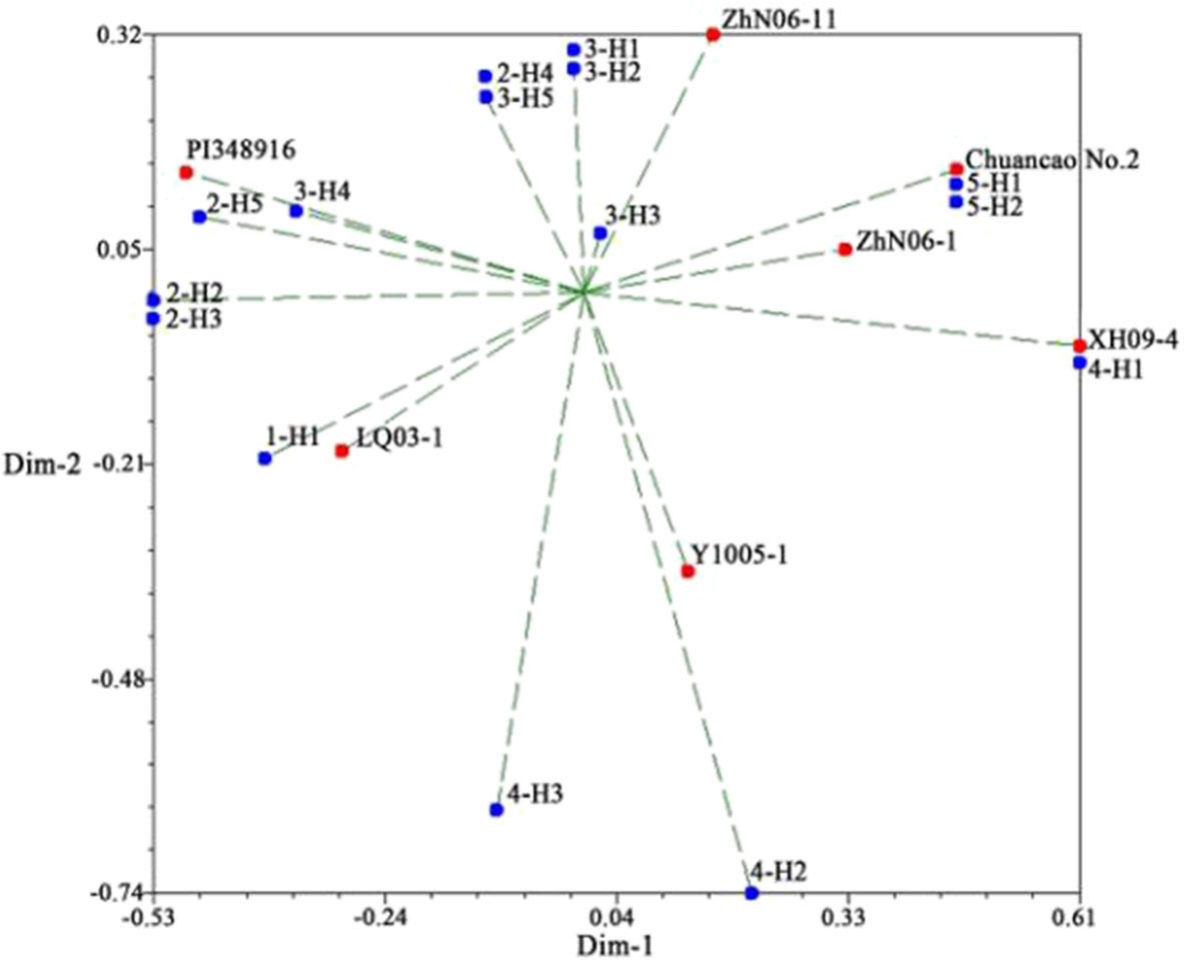
Principal coordinates analysis for EST-SSR markers using genetic similarity matrix for 22 *E. sibiricus* accessions. Red dot and blue dot represented parents and hybrid populations, respectively

The principal coordinate analysis (PCoA) showed about 64.72% of the total variation was described by the first three PCo (Fig. 3). The results of PCoA analysis were similar to structure analysis.

## Discussion

### EST-SSR markers for hybrid identification

Traditional hybrid identification depends on morphological traits like plant height, leaf shape, flower color, growth habit, and so on. But environmental factors often affect the accuracy of identification. DNA fingerprinting techniques are efficient alternatives to morphological identification, and make plant identification easy and accurate. Molecular markers have been successfully applied to identification of crop and forage grass species [25, 26]. EST-SSRs are codominant markers that can show evidently the heterozygosity of offspring populations by the bands from parents and hybrid plants [10, 27]. Our study is the first report of *E. sibiricus* hybrids identification using EST-SSR markers. Based on our results single primers or primer combinations that were diagnostic for parental plants and hybrid plants could be used for hybrid identification. Fifteen hybrids were identified with two types of DNA fingerprinting. In general, this study demonstrated that EST-SSR markers can effectively identify *E.sibiricus* hybrids through special DNA fingerprinting profiles. This study will lay a foundation for *E. sibiricus* breeding in future.

### EST-SSR marker for genetic diversity in *E. sibiricus*

EST-SSRs are highly polymorphic, abundant and are accessible to research laboratories via published primers sequences [4]. These published primers are especially important resources for species like *E. sibiriucs* with few molecular markers available. In this study, a total of 9 EST-SSR markers from three different genuses were used for hybrid identification and genetic diversity analysis. The transferability rate of EST-SSRs was 100%. Results of this study were consistent with previous reports that EST-SSR markers have high transferability rate among species. Information on genetic diversity and hybrid population and their parents can improve our understanding of breeding materials.Based on our results the percentage of polymorphism varied from 65.12 (Pop 5) to 75.68% (Pop 1), with an average of 70.51%. The average percentage of polymorphism (PPB) of five hybrid populations were lower than previous reports of SRAP (PPB = 86.5%) [28], SSR (PPB = 89.4%) [4], and SCoT (PPB = 91.9%) [29]. The major reason for relatively low PPB could be small sample size of each population. In addition, hybrid populations derived from seven parent genotypes, and genetic base was relatively narrow. This can be supported by results of structure analysis. In general, these accessions and their offspring did not show major genetic structure, most of accessions were assigned into mixed groups, indicating relatively narrow genetic base.

### Broadening genetic diversity for breeding

In this study, some types of polymorphism have evidenced potentially an occurrence of chromosome variation or gene rearrangements in hybrid populations that resulted from the gain of novel bands. The results of the present study suggested that more genetic diversity and new variation could be captured by crossing breeding. Whether these novel bands were responsible to new genes associated to seed shattering or other important traits, it was still not clear. It is still difficult to address particular mechanisms understanding the chromosomal or genomic rearrangements in response to novel bands. In the future, molecular markers combined with sequence data might provide evidence to the inheritance of transcribed regions and gene functions.

## Abbreviations

EF1: Bands exclusively present in hybrids
FF1: Bands shared by female parents and hybrids
M: Number of monomorphic bands
MF1: Bands shared by male parents and hybrids
PCoA: Principal coordinate analysis
PPB: Percentage of polymorphic bands
T: Total number of amplified bands
TP: Total number of polymorphic bands
